# Molecular docking analysis of piperlongumine with different apoptotic proteins involved in Hepatocellular Carcinoma

**DOI:** 10.6026/97320630017829

**Published:** 2021-09-30

**Authors:** Ashish Kumar, Ambika Sharma, Shailendra Handu, Jagjit Singh, Manisha Naithani

**Affiliations:** 1 Department of Biochemistry, All India Institute of Medical Sciences (AIIMS), Rishikesh-249203, Uttarakhand, India; 2 Department of Biochemistry, College of Veterinary Science, DUVASU, Mathura-281001, Uttar Pradesh, India; 3 Department of Pharmacology, All India Institute of Medical Sciences (AIIMS), Rishikesh-249203, Uttarakhand, India

**Keywords:** Hepatocellular Carcinoma, Piperlongumine, Vascular Endothelial Growth Factor, Molecular Docking, AutoDock

## Abstract

Hepatocellular carcinoma (HCC) is the most common type of primary liver cancer. Numerous signalling pathways are involved in hepatocellular carcinoma. Piperlongumine is a potential candidate for the treatment of hepatocellular carcinoma. Therefore, it is of interest to document the molecular docking analysis of piperlongumine with different apoptotic proteins involved in Hepatocellular Carcinoma. Piperlongumine was docked with the HCC targets such as vascular endothelial growth factor (VEGF), epidermal growth factor receptor, Aurora-2, Nuclear factor Kappa-B (NF-KB), Jak2 Kinase, Fibroblast growth factor receptor 4, Bcl-2-like protein 1,Apopain, and Apoptosis regulator Bcl-2 using *in-silico* technique with the software grid-based ligand docking with energies. Piperlongumine exhibited the highest negative energy value (E-value) of -6.58 kcal/mol with vascular endothelial growth factor receptor 2, followed by -5.46, -5.34, -5.31, and -5.29 kcal/mol with 1M17, 2BMC, 1SVC, 4C61, 4XCU with epidermal growth factor receptor, aurora-2, nuclear factor Kappa-B (NF-KB), Jak2 kinase, and fibroblast growth factor receptor 4 (FGFR4), respectively for further consideration.

## Background:

Hepatocellular carcinoma (HCC) is one of the leading causes of death in patients with liver cancer. It is one of the most common cancers and the third leading cause of cancer-related death worldwide [1]. It remains a lethal disease with poor prognosis in patients [2]. According to population-based registries, the crude incidence rate of HCC in India, in 2015, was 2.8 cases per 100,000 people per year (males: 3.9, females: 1.6), and the crude mortality rate was 2.7 per 100,000 people per year [3]. HCC is the seventh most common cause of cancer-related death in India. Patients with advanced HCC have a poor prognosis with a reported median survival of only 2–3 months with the best supportive care [4]. HCC commonly develops in patients having chronic infection with hepatitis B (HBV) or hepatitis C virus (HCV), aflatoxin contaminated foods, heavy alcohol intake, obesity, type 2 diabetes, and smoking [5]. Sorafenib, a multikinase inhibitor, is a standard drug of choice for the treatment of HCC; however, the therapeutic window with sorafenib is narrow [6].

Piperlongumine (PL), is an amide alkaloid isolated from Piper longum. It is widely used in Indian traditional medicine, and has been found to have potential anticancer effects in HCC [7]. Piperlongumine exert its anticancer effect by various mechanism that includes ROS accumulation only in cancer cells, cell cycle arrest, down-and up-regulation of various proteins (GSTP1,VEFG), apoptosis and modulating key regulatory proteins,including PI3K, AKT, mTOR, NF-kβ, STATs, and cyclin D1 [8-11]. Molecular Docking is an important part of computer-aided drug discovery. It aids in the prediction of the intermolecular framework formed by a protein and its ligand, and yields the appropriate binding between the molecules. Signaling pathways have become a major basis of targets in HCC [12]. Therefore, it is of interest to document the molecular docking analysis of piperlongumine with different apoptotic proteins involved in Hepatocellular Carcinoma.

## Methodology:

### Protein Preparation:

Protein Data Bank (RCSB PDB) with PDB IDs was used to retrieve the crystal structures of target proteins and was carried further for more studies of docking process. The crystal structure of 2BMC, 4C61, 4XCU, 1SVC, 4QVF, 2OH4, 4ASD, 1M17, 2O2F, and 1CP3 receptor subunit was downloaded from PDB database. All the non-essential water molecules were removed from the crystal structure of 2BMC, 4C61, 4XCU, 1SVC, 4QVF, 2OH4,4ASD, 1M17, 2O2F, and 1CP3 receptor.

### Ligand preparation:

The ligand structure was obtained from the PubChem database and converted into three-dimensional structure (3D) before analysis in SDF format, converted further to PDB format using PyMOL software. Metals were also removed from the ligands using PyMOL software in order to conduct appropriate docking studies. The prepared ligands were saved in PDB format for further docking studies. Based on energy minimization, the drug binds to effectors/receptors in the most stable form that is the minimum energy form. The active compounds were subjected to conformational analysis and energy minimization using Monte Carlo conformational search. Low energy conformers of all the structures were generated, and utilized further for analysis [14].

### Molecular docking:

Following the receptor and ligand preparation, AutoDock tool was used for the protein synthesis and grid generation. Docking was performed by AutoDock 4.2.6 program, using the implemented empirical free energy function and the Lamarckian Genetic Algorithm (LGA). Polar hydrogens were added into the structure and Gasteiger charges were calculated and applied consequently. Missing residues in the proteins were also added at the time of preparation. Molecular docking study of piperlongumine and 2BMC, 4C61, 4XCU, 1SVC, 4QVF, 2OH4,4ASD, 1M17, 2O2F, and 1CP3 were executed with AutoDock 4.2 software. The grid maps were calculated using AutoGrid. In all dockings grid-point spacing of 1.000 Å was applied. Using the gradient optimization algorithm and an empirical scoring function, the molecular docking was conducted to generate the best binding affinity or fitness of protein-ligand binding poses between compounds as 2BMC, 4C61, 4XCU, 1SVC, 4QVF, 2OH4,4ASD, 1M17, 2O2F, 1CP3 receptor and piperlongumine ligand. The best binding conformations of ligands were selected and analyzed using AutoDock 4.2 software as well as in Discovery Studio 4.0 [15]. The best conformation with the lowest docked energy was chosen from the docking search. Number of torsions are choosen from 0-6, and if any ligand shows more than 6 it is adjusted to 6. Hydrogen bond interactions are also calculated and mentioned, presence of H-bonds indicates a stable interaction between the ligand and the protein. Discovery studio 2020 Client and Chimera softwares are used to depict hydrogen bonds, 2-D images and protein-ligand interactions images for a good visualization of the docking.

## Results and Discussion:

Hepatocellular carcinoma is one of the most common cancers worldwide. In this study, we used a molecular docking protocol and predicted the potential targets of piperlongumine among liver proteins from the Protein Data Bank. The goal of molecular docking is to predict the structure of the ligand-receptor complex using computation methods. Molecular docking was performed to obtain more insights about interactions between the protein 2BMC, 4C61, 4XCU, 1SVC, 4QVF, 2OH4, 4ASD, 1M17, 2O2F, and 1CP3 with piperlongumine ligand. Molecular docking study was carried out by AutoDock 4.2 software, using the implemented empirical free energy function and the Lamarckian Genetic Algorithm (LGA). Table 1(see PDF) displays all the data on binding energies (kcal/mol), the number of hydrogen bonds, and the number of closest residues around the active site. Molecular docking score of 2BMC, 4C61, 4XCU, 1SVC, 4QVF, 2OH4, 4ASD, 1M17, 2O2F, and 1CP3 were found to be -5.34, -5.29, -5.23, -5.31, -4.92, -6.58, -5.09, -5.46, -5.13, and -4.75 respectively. Piperlongumine shows one hydrogen bond with ALA213 amino acid with 2BMC receptor, with THR830 amino acid with 1M17 receptor and GLU1006 amino acid with 4C61 receptor, three hydrogen bonds with TYR642, ILE640, and ARG664 amino acid with 4XCU receptor. Piperlongumine shows one hydrogen bonds with GLU233, LEU45 amino acids with 1SVC receptor and one hydrogen bonds with ASN136, ARG139 amino acids with 4QVF receptor. It shows two hydrogen bonds with ASP1046, LYS868 amino acids with 4ASD receptor and with HIS121, GLY122 amino acid with 1CP3 receptor. 3D and 2D docking images of 2OH4 (vascular endothelial growth factor-VEGFR2 is represented in Figure 1(see PDF). Binding energy is a measure of the ligand-protein complex affinity, or the difference between the energy of complex and the sum of energies of each molecule separately [16]. The more less binding energy seen for protein 2OH4 is -6.58 then for 1M17 IS -5.46, 2BMC is -5.34, 4C61 is -5.29, 4XCU is -5.23 and 2O2F if -5.13 and so on. The present study suggests that among the various liver cancer protein 9 compounds docked with piperlongumine and showed the best interaction with 2OH4,1M17, 2BMC, 1SVC, 4C61, 4XCU with binding energy more than -5 kcal/mol. Piperlongumine had the most effective interaction with Vascular endothelial growth factor (VEGF), epidermal growth factor receptor (EGFR), Aurora-kinase, Nuclear factor Kappa-B (NF-KB), and Jak2 Kinase. Protein–ligand interaction plays a major role in identification of the possible mechanism by which a ligand can bind with the target and exerts the pharmacological action [17]. It has been demonstrated that piperlongumine blocked NF-κB activated by TNFα and this inhibition in NF-κB activity downregulated the expression of proteins involved in cell survival (Bcl-2), and invasion (VEGF) [18]. PL inhibited collagen-induced platelet reactivity by targeting the JAK2-STAT3 pathway [19]. The binding efficiency of piperlongumine with the all ten liver cancer protein were good. The molecular docking studies between liver cancer proteins and piperlongumine clearly demonstrated the mode of binding and interacting active site amino acids between them and the hydrogen bond interaction of piperlongumine with liver cancer protein. Piperlongumine was found to bind the liver cancer protein 1CP3 with least free energy less than compared other liver cancer protein, it may probably activate apoptotic proteins in carcinoma thereby acting as potent anticancer agent.

## Conclusion:

We document the molecular docking analysis of piperlongumine with different apoptotic proteins involved in Hepatocellular Carcinoma. Data shows optimal binding features of piperlongumine with CYP1A2 for further consideration in this context.
